# Rapid target validation in a Cas9-inducible hiPSC derived kidney model

**DOI:** 10.1038/s41598-021-95986-5

**Published:** 2021-08-16

**Authors:** Yasaman Shamshirgaran, Anna Jonebring, Anna Svensson, Isabelle Leefa, Mohammad Bohlooly-Y, Mike Firth, Kevin J. Woollard, Alexis Hofherr, Ian M. Rogers, Ryan Hicks

**Affiliations:** 1 Translational Genomics, Discovery Sciences, BioPharmaceuticals R&D, AstraZeneca, Gothenburg, Sweden; 2 Quantitative Biology, Discovery Sciences, BioPharmaceuticals R&D, AstraZeneca, Cambridge, UK; 3 Bioscience Renal, Research and Early Development Cardiovascular, Renal and Metabolism, BioPharmaceuticals R&D, AstraZeneca, Cambridge, UK; 4 Early Clinical Development, Research and Early Development, Cardiovascular, Renal and Metabolic, BioPharmaceuticals R&D, AstraZeneca, Gothenburg, Sweden; 5 Department of Physiology, University of Toronto, Toronto, ON M5S 1A8 Canada; 6 Lunenfeld-Tanenbaum Research Institute, Mount Sinai Hospital, Toronto, ON M5G 1X5 Canada; 7 Soham & Shaila Ajmera Family Transplant Centre, University Health Network, Toronto, ON M5G 2C4 Canada; 8 Department of Obstetrics and Gynecology, University of Toronto, Toronto, ON M5G1E2 Canada; 9 BioPharmaceuticals R&D Cell Therapy, Research and Early Development, Cardiovascular, Renal and Metabolism (CVRM), BioPharmaceuticals R&D, AstraZeneca, Gothenburg, Sweden

**Keywords:** Genetic engineering, Target identification, Target validation, Pluripotent stem cells, Stem-cell differentiation, Kidney

## Abstract

Recent advances in induced pluripotent stem cells (iPSCs), genome editing technologies and 3D organoid model systems highlight opportunities to develop new in vitro human disease models to serve drug discovery programs. An ideal disease model would accurately recapitulate the relevant disease phenotype and provide a scalable platform for drug and genetic screening studies. Kidney organoids offer a high cellular complexity that may provide greater insights than conventional single-cell type cell culture models. However, genetic manipulation of the kidney organoids requires prior generation of genetically modified clonal lines, which is a time and labor consuming procedure. Here, we present a methodology for direct differentiation of the CRISPR-targeted cell pools, using a doxycycline-inducible Cas9 expressing hiPSC line for high efficiency editing to eliminate the laborious clonal line generation steps. We demonstrate the versatile use of genetically engineered kidney organoids by targeting the autosomal dominant polycystic kidney disease (ADPKD) genes: *PKD1* and *PKD2*. Direct differentiation of the respective knockout pool populations into kidney organoids resulted in the formation of cyst-like structures in the tubular compartment. Our findings demonstrated that we can achieve > 80% editing efficiency in the iPSC pool population which resulted in a reliable 3D organoid model of ADPKD. The described methodology may provide a platform for rapid target validation in the context of disease modeling.

## Introduction

Chronic kidney disease (CKD) affects more than 10% of the global population and causes substantial mortality and economic burden^1,2^. More work is needed to recognize the underlying causes in a large proportion of patients with CKD^2^. More than 25% of patients with CKD report a family history^3^ and above 200 causative single gene mutations are suggested to predispose to CKD^4^. Nevertheless, known genetic causes of CKD represent only a few of the total number of patients with CKD^5^. In addition, genome-wide association studies have identified a dozen genomic regions harboring the probable causal mutations, which require further functional validation^6^. Moreover, owing to the decline in the high-throughput sequencing costs, several ambitious large-scale genome programs aiming at establishing high quality genetic variant databases are either completed or initiated across the globe. For example, 66 different monogenic disorders were detected in a recently published study^7^, in which exome sequencing was used in a combined cohort of more than 3000 patients with CKD^7^. However, those studies highlight the need for translatable disease models for validation of the newly discovered mutations in order to accelerate the drug discovery process.

The terms “Target Identification (TI)” and “Target Validation (TV)” refer to key steps in drug discovery programs that determine and confirm (i) an association between a gene and a disease and (ii) modulating the identified gene is likely to ameliorate disease phenotype in patients^8,9^. The demand for improved TV platforms grows as the number of identified gene-disease associations increases. Ideally, a TV platform (e.g., an in vitro cellular assay) should functionally validate the mutations in their genomic, cellular and human-specific contexts at high speed and capacity to enable high-throughput programs. Given the cellular complexity of the kidney, the lack of disease models that can be used as reliable TV platforms have remained a challenge for decades. However, advances in direct differentiation of human induced pluripotent stem cells (hiPSCs) into three-dimensional (3D) organoids and RNA-guided gene-targeting technologies hold great promises for overcoming these challenges.

An organoid is a complex in vitro 3D multicellular tissue derived from different cell types which resembles the structure and the functionality of the real organ in humans^10^. Remarkable progress has been achieved during the last decade in shifting towards the use of organoids for modeling human diseases^11,12^. Organoid models for many tissues e.g., brain, gastrointestinal tract, breast and liver have now been reported. Likewise, several protocols have been described in the last five years for generation of kidney organoids^13–17^. Notably, our team has previously reported on developing kidney organoids for drug discovery applications^15^. Along with the progress in generation of human 3D models, a revolutionary gene targeting technology, the CRISPR-Cas9 system, has rapidly been established for genome manipulation. CRISPR (Clustered, Regularly Interspaced, Short Palindromic Repeats)-Cas9 system is originally harnessed from bacterial adaptive immune system^18^ for applications in human cells^19,20^. This technology encompasses an endonuclease protein (Cas9) and an RNA molecule, designated as short guide-RNA (sgRNA) guiding Cas9 to the specific target locus in the genome. Different genomic sites can be targeted by providing different sgRNA molecules that match the targeted sites. The Cas9-induced DNA breaks often result in the formation of small insertion or deletion (indels) that can subsequently cause frameshifts within the coding sequence, thereby yielding truncated or non-functional proteins. CRISPR-Cas9 unequivocally provides scientists with a tool to study gene functions by comparing wild-type and edited phenotypes.

Applying both 3D kidney differentiation and CRISPR technologies, is opening new opportunities for researchers to study the genetic basis of kidney disorders with an unprecedented translatability. However, notable challenges still exist. One hurdle to increase the throughput of gene knockout and phenotypic studies in kidney organoids is the need for the generation of clonal knockout cell lines followed by their differentiation into organoids. This process is time consuming and labor-intensive, requiring several months for validation of the knockout line. Hence, more work is needed in exploring different routes to improve this lengthy process. Here, we report the successful use of high efficiency knockout pool populations for studying kidney disease phenotypes. This allowed us to skip the generation of clonal cell lines and establish a speedy target validation platform.

## Results and discussion

In this study, we selected a doxycycline-inducible Cas9 expressing hiPSC line^21^ to conduct gene-editing and differentiation experiments. The reason behind selecting the Cas9-inducible cell line in our study was to try and achieve as high a rate of editing as possible, as each cell would express Cas9 upon doxycycline induction and so only guides would need to be introduced to the cells to enable efficient editing, simplifying the transfection step. This cell line contains a Cas9 expressing cassette integrated into the AAVS1 locus using the ObLiGaRe system^21,22^.

**Figure 1:**
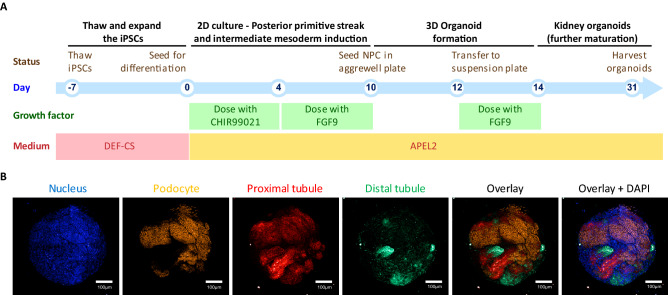
Differentiation of hiPSC into kidney organoids. (**A**) The schematic outline for the multi-stage kidney organoid differentiation is shown. (**B**) The resulting kidney organoids (day31) stained and imaged using confocal microscopy for DAPI and kidney specific markers including WT1, LTL and ECAD for podocyte, proximal tubule and distal tubule, respectively.

For organoid development, we optimized a previously published kidney organoid differentiation protocol^15^ on this cell line to validate it’s competency for differentiation to kidney organoids. This protocol consisted of two stages. In the first stage cells were cultured in conventional plates for 10 days; and media were supplemented with CHIR99021 and FGF9 to initiate differentiation. This resulted in the generation of kidney progenitor cells. In the second stage, cells were transferred into aggrewell plates to allow formation of organoids. Finally, organoids were transferred into suspension plates for further maturation until day 31 when they were harvested for phenotyping studies. A schematic presentation of our kidney organoid protocol is presented in Fig. 1A.

The inducible Cas9 hiPSC line subjected to our kidney differentiation protocol resulted in the development of nephron-like structures in each organoid. The resulting kidney organoids were positive for markers of podocyte (WT1), proximal tubules (LTL) and distal tubules (ECAD) (Fig. 1B). Analysis of max projection images from 10 separate differentiation experiments, measuring marker positive areas, showed that we typically see an average of 10–15% distal tubuli (ECAD), 5–10% podocytes (WT1) and 10–20% proximal tubuli (LTL) in our kidney organoids (data not shown).

**Figure 2:**
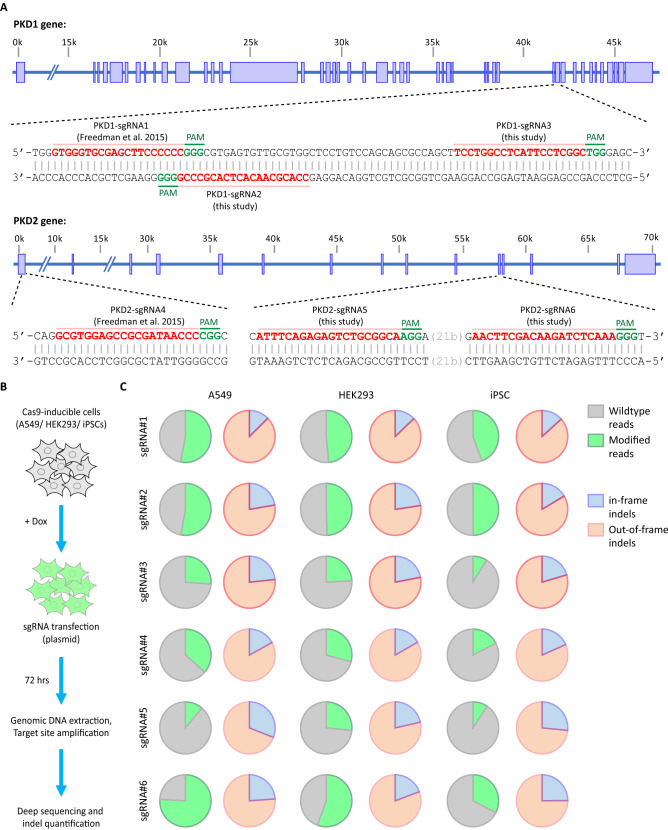
Validation of sgRNAs against *PKD1* and *PKD2*. (**A**) Schematic presentation of the target sites from previous report^23^ and our study is shown. (**B**) Outline of the experimental procedure used for validation of the sgRNAs. Cells were seeded in 96-well format and Cas9 expression was induced before transfection with sgRNA-expressing plasmids. (**C**) The resulting Cas9-induced mutations at the target sites were measured using deep targeted sequencing. Mutations were further analyzed for their effect on the reading frame.

Next, we used Cas9-mediated gene editing to introduce mutations in *PKD1* and *PKD2*. These two genes were selected because mutations in these genes cause ADPKD and can therefore serve as positive controls (i.e., expecting the disease phenotype) for evaluation of our methodology. ADPKD is an inherited monogenic renal disease characterized by the accumulation of clusters of fluid-filled cysts in the kidneys. Previous studies have confirmed that knockouts of *PKD1* or *PKD2* result in formation of a cyst-like phenotype in kidney organoids. For example, Freedman and colleagues reported an in vitro model for polycystic kidney disease (PKD) by introducing biallelic mutations in *PKD1* or *PKD2* using CRISPR-Cas9 and generating kidney organoids from a clonal knockout iPSC line. Those knockout organoids formed cyst-like structures in kidney tubules, recapitulating some characteristic features of the disease phenotype in vitro^23,24^. In our study, we evaluated the activity of the previously described *PKD1* and *PKD2* sgRNAs (PKD1-sgRNA1 and PKD2-sgRNA4 respectively), in addition to new sgRNAs designed in this study for *PKD1* (PKD1-sgRNA2 and PKD2-sgRNA3) and *PKD2* (PKD1-sgRNA5 and PKD2-sgRNA6) (Fig. 2A). In brief, to test the activity of the sgRNAs, we first transfected the sgRNA-expressing plasmids into the Cas9-induced cells and performed deep sequencing on the targeted loci at 72 h post transfection. All sgRNAs showed activity in all three different human cell lines (HEK293T, A549, hiPSCs) tested in our study (Fig. 2B). We could detect mutations introduced at the targeted sites using deep targeted sequencing, with efficiencies ranging from approximately 15–75% and of these mutated sequences, around 70–80% of these mutations caused frameshifts (Fig. 2C).

**Figure 3:**
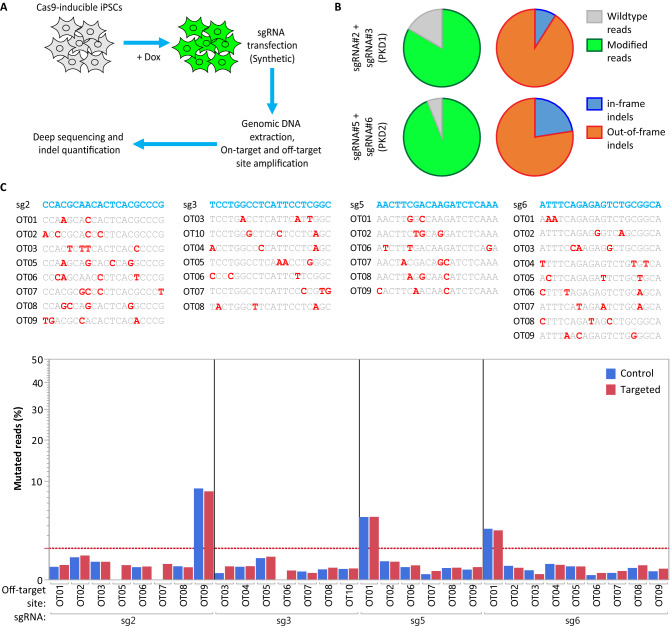
Paired-sgRNA strategy resulted in higher indel rate in hiPSCs. (**A**) Schematic outline of the experiment is shown. (**B**) The percentage of the modified reads for on-target loci were measured using deep targeted sequencing. (**C**) No Cas9 off-target activity was detected at the selected off-target sites. Samples were analyzed using deep targeted sequencing.

The editing efficiency of the tested sgRNAs were higher in HEK293T and A549 cell lines than in hiPSCs, presumably due to the different transfection efficiencies. However, the normalized percentage of the frameshifting mutations were consistent among different cell types regardless of the variations in the achieved editing efficiencies (Fig. 2C). In hiPSCs, the highest editing efficiencies were about 50% and 30% for *PKD1* and *PKD2* genes, respectively. These numbers represent the number of mutated alleles in the pool of cells and do not represent the percentages of the cells that contain mutations on both alleles. To achieve a higher genome-editing efficiency, we rationalized that paired sgRNAs would result in a higher indel formation than single guides alone. This approach is based on the proximity of two sgRNAs introducing two simultaneous DNA breaks. It has been previously shown that the repair at these DNA breaks is mediated by Non-Homologous End Joining (NHEJ) repair pathway and results in the precise deletion of the DNA sequence between the two cut sites^25^. Additionaly, we tested synthetic-sgRNA transfection into the Cas9-induced hiPSCs instead of plasmid DNA transfection (Fig. 3A). Introducing these changes resulted in achieving a remarkable > 80% editing efficiencies in both *PKD1* and *PKD2* using deep targeted sequencing (Fig. 3B). Next, we examined the off-target sites for all tested sgRNA-pairs by performing deep targeted sequencing. Prospective off-target sites were identified using an in-house developed software identifying sites with up to a maximum of four mismatches. Our results showed no Cas9 off-target activity at any of these evaluated loci (Fig. 3C). Investigating Cas9 off-target effects is an important aspect of performing gene editing experiments as previous reports have shown that some off-target sites can be edited at a similar efficiency as the on-target site^26^.

We further examined the *PKD* knockout pools (PKD KOp) for pluripotency and differentiation capability after CRISPR-Cas9 gene editing. PKD KOp iPSCs expressed expected pluripotency markers such as octamer-binding transcription factor 4 (OCT4) and NANOG (Supplementary Fig. S1a). The tri-lineage differentiation potential was assessed using immunofluorescence analysis of markers for ectoderm (PAX6 and Nestin) (Supplementary Fig. S1b), endoderm (SOX17 and CXCR4) (Supplementary Fig. S1c) and mesoderm (Brachyruy and CXCR4) (Supplementary Fig. S1d) along with DAPI for nucleus staining in 2-dimensional cultures. As expected, our results showed that *PKD1* and *PKD2* are dispensable for pluripotency and differentiation potential. These observations are in line with the previous report^27^.

Next, we confirmed that mutations in iPSC cells could be maintained across multiple passages and during differentiation. To achieve this, samples were collected at different time points and the editing frequency analyzed on the pool of cells (Supplementary Fig. S1e). We observed similar editing frequency (80–90%) across all collected samples, indicating that the edited cells were not depleted during passages.

**Figure 4:**
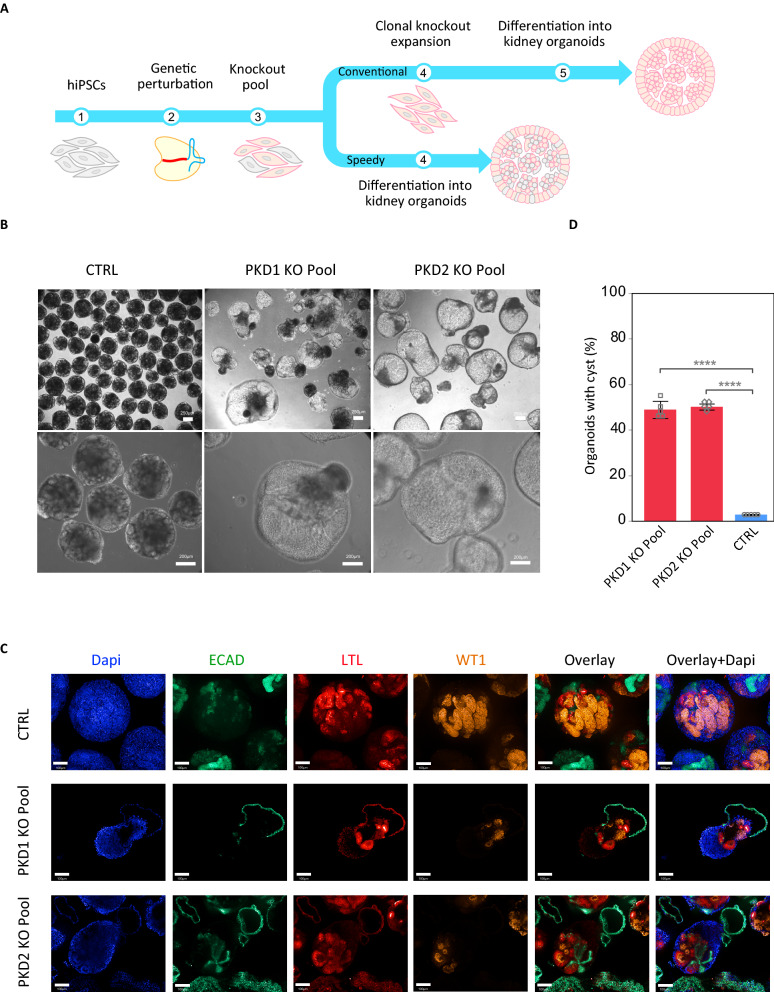
Cystic phenotype in kidney organoids using genetic perturbation. (**A**) Schematic outline of differentiation stages for conventional clonal knockout isolation and differentiation and speedy/direct differentiation of KO pools. (**B**) Cystic phenotype observed in organoids obtained from genetic perturbation of *PKD1* and *PKD2*. (**C**) The resulting kidney organoids were stained and imaged using confocal microscopy for different markers. (**D**) Quantification of cyst formation rates in PKD KO pool organoids versus isogenic unmodified organoids (CTRL) as an average of all experiments. Data are shown as Mean. Bar represent standard deviation (SD) (n = 5 separate experiments).

We then evaluated whether *PKD* knockout pools (PKD KOp) could be differentiated into kidney organoids to obtain the previously observed cystic phenotype^23,24^. Both PKD KOp hiPSCs and isogenic unmodified cells (control) were differentiated into kidney organoids (Fig. 4A). We could detect the expected formation of fluid-filled cyst-like structures in about 50% of the PKD KOp organoids but at a very low rate (less than 1%) in our control organoids (Fig. 4B–D). Achieving an efficient gene-editing frequency (> 80%) therefore eliminates the need for laborious clonal cell line isolation. This is an advantage for target validation studies where large sets of genetic factors need to be experimentally tested.

Taken together, we described a highly tractable 3D kidney organoid model that consistently recapitulated ADPKD cystogenesis in vitro. Going forward, this organoid model of ADPKD may provide a high-throughput opportunity to study human cyst initiation and modification in a disease-relevant multicellular model.

## Conclusion

In this study we demonstrated that efficient genome editing could be achieved using an inducible CRISPR-Cas9 expressing iPSC cell line to speed up generation of genetically-modified kidney organoids. Our results are useful in an industrial settings where large scale experiments are desired to screen reliable phenotypes for identification of targets for initiating drug discovery programs. In conventional settings, the need for generation of the clonal lines is a major bottleneck in scalability of the target identification and target validation studies. By using knockout pools instead of generating isogenic clonal lines, our approach would enable faster turnaround time by using knockout pools instead of isogenic clonal lines. Moreover, our work presents a novel disease model for ADPKD target validation.

## Experimental procedures

### Cell culture

All experiments presented in this study used the previously described doxycycline-inducible Cas9 expressing hiPSC line^21^. Undifferentiated hiPSCs were maintained and transfected in the feeder-free and chemically defined culture system DEF-CS 500 (Cellartis by Takara Bio Europe). Cells were passaged every three to four days using TrypLE Select (Gibco) and single cells reseeded onto fresh DEF-coated plate using DEF-CS media with 10 µM Rock inhibitor (Tocris bioscience). Quality control of the cells was based on morphology, karyotype and pluripotency characteristic of the line. Doxycycline-inducible Cas9-expressing 293 T and A459 cells were cultured and transfected in DMEM-glutamin (Gibco) supplemented with 10% fetal bovine serum (Gibco) and 1% penicillin/streptomycin (Gibco). All cells were maintained at 37 °C in a humidified 5% CO_2_ incubator.

### Kidney organoid differentiation

CRISPR-targeted and non-mutant isogenic control hiPSCs were differentiated into kidney organoids using a modified version of the protocols published by Takasato et al^16,28^ and Morizane et al^17^ involving inhibition of glycogen synthase kinase-3B (GSK3β) and stimulation by FGF9. The day before differentiation, hiPSCs were dissociated into single cells from 100% confluent cultures with TrypLE Select (Gibco) and plated at 15,000 cells/cm^2^ in a 6-well plate pre-coated with Geltrex Reduced Growth Factor Basement Membrane Matrix (Gibco) in DEF-CS 500 medium with 10 µM Rock inhibitor. Cells were incubated overnight at 37 °C and 5% CO_2_. On the next day cells were rinsed with PBS^−/−^ and treated with 6 µM CHIR99021 (Tocris Biotechne) in APEL2 basal medium (STEMCELL Technology) supplemented with 5% Protein Free Hybridoma Media (PFHM -II; Gibco) and 1% penicillin/streptomycin (Thermo Fisher Scientific) for 4 days, followed by 5 days with FGF9 (200 ng/ml), during which medium was changed every second day. At day 10, cells were dissociated into single cells using TrypLE select, plated at 1.2 × 10^6^ cells per well in AggreWell400 (Stemcell Technologies) and treated with 3 µM CHIR99021, 200 ng/ml FGF9 and 10 µM Rock inhibitor in APEL2 basal medium. Plates were centrifuged at 200*g* for 15 s at room temperature and incubated at 37 °C and 5% CO_2_ for 2 days. After 2 days (day 12), organoids were transferred to ultra-low adhesion plates in a shaker incubator (at 37 °C and 5% CO_2_) rotating at 100 rpm. On days 12 and 14 organoids were treated with 200 ng/ml FGF9 in APEL2 basal medium. From day 14 onwards, suspension culture continued in factor-free APEL2 basal medium for up to 17 days, with medium change three times per week.

### Immunofluorescence and confocal microscopy

Kidney organoids were collected in a 1.5 ml microtube, washed with PBS^−/−^ before being fixed in 4% PFA (Ninolab) for 30 min to 1 h at room temperature. Organoids were then washed three times with PBS^−/−^ for 1 h and stored in PBS^+/+^ at 4 °C until staining. Fixed organoids were permeabilized and blocked with 5% normal FBS (Life Technologies) in PBS^−/−^ with 0.3% Triton X-100 (Sigma-Aldrich) for 3 h at room temperature. Primary antibodies were added overnight at 4 °C in a solution of PBS^−/−^ with 0.5% FBS and 0.5% Triton X-100 (wash buffer). Unbound primary antibodies were washed in wash buffer three times for 3 h. Species-matched Alexa-Fluor secondary antibodies (Invitrogen 1:500 dilution) and Hoechst (Thermo Fisher Scientific, 1:1000 dilution) were diluted in wash buffer and added overnight at 4 °C. Samples were washed one last time with PBS^−/−^ for 3 h before imaging. The images were taken using CV7000 confocal microscope. The primary antibodies used were WT1 (Abcam ab89901, 1:300), ECAD (Abcam ab11512, 1:200) and LTL (Vector Lab B-1325, 1:300).

### Pluripotency analysis

Human iPSCs (PKD KO and isogenic control) were fixed and stained for pluripotency markers OCT4 (Milipore mab4401,1:200), and NANOG (Stemgent 09-0020, 1:100) as described above.

### Trilineage differentiation potential

Human iPSCs (PKD KO and wildtype control) were differentiated into the 3 germ layers according to the STEMdiff Trilineage Differentiation Kit protocol (STEMCELL Technologies). Briefly, cells were seeded onto Matrigel-coated plates and treated with endoderm and mesoderm medium for 4 days and ectoderm medium for 6 days. Differentiated cells were then fixed and stained with antibodies against lineage-specific markers for ectoderm, endoderm and mesoderm as described above. Primary antibodies used were CXCR4 (R&D system mab173, 1:500), SOX17 (R&D system af1924, 1:20), Brachyury (R&D system af2085, 1:100), Nestin (R&D system mab1259, 1:500) and PAX6 (Stemgent 09-0075, 1:200).

### CRISPR-Cas9 design and generation

Paired-sgRNAs targeting exon 36 of *PKD1* (5′- CCACGCAACACTCACGCCCG -3′, 5′-TCCTGGCCTCATTCCTCGGC-3′) and exon 11 of *PKD2* (5′-ATTTCAGAGAGTCTGCGGCA-3′, 5′-ATTTCAGAGAGTCTGCGGCA-3′) were designed. Plasmid guide RNA was generated by cloning targeting oligos into a U6 promoter-driven backbone vector using digestion ligation cloning. Chemically synthesized oligoribonucleotides were manufactured by Synthego.

### sgRNA transfection

hiPSCs were treated with Doxycycline (10 µg/ml) for 1 h. Cells were washed three times with PBS^−/−^. Transfection was done 24 h post-induction using CRISPRMAX (Invitrogen).

HEK293T and A549 cells were treated with doxycycline (100 ng/ml) overnight. Transfection was done the next day using FuGene HD (Promega). All experiments were performed in triplicate.

### Deep targeted amplicon sequencing

Genomic DNA was isolated either using Puregene cell and tissue kit (Qiagen) or QuickExtract DNA Extraction Solution (Lucigen, Epicenter) according to the manufacturer’s recommended protocols. Gene specific primers were designed using Primer-BLAST tool^29^. Targeted genomic sites were first amplified using gene-specific primers linked with the Illumina Nextera adapters (listed in Supplementary Table S1) using PhusionFlash High-Fidelity PCR MasterMix (Thermo). Amplifications were performed either using 100 ng of genomic DNAs extracted with PureGene kit or using 2 µl of the cell lysate in 20 µl PCR containing 0.25 µM final concentration of each forward and reverse primers. The following thermal cycling condition was used in the first PCR: 1 cycle initial denaturation (2 min at 98 °C), 35 amplification cycles (10 s at 98 °C, 10 s at 60 °C, 5 s at 72 °C) and 1 cycle final extension (5 min at 72 °C). Amplicons then were purified with Agencourt AMPure XP beads (Beckman Coulter) according to the manufacturer’s recommended protocol and concentrations were measured using Fragment Analyzer (Agilient’s Advanced Analytical Technologies). A secondary indexing PCR was performed using Illumina Indexes followed by a purification and concentration measurement as previous described. The indexed libraries were pooled and subjected to paired-end sequencing using mid-throughput 300 cycles (2 × 150 cycles) on an Illumina NextSeq500 instrument. Sequencing reads were analyzed as described previously^30^. Briefly, sequencing reads were quality controlled and merged using FLASH software^31^. Reads were mapped to the amplicon reference sequence using bwa software^32^. Variants (excluding the single nucleotide variations) were called with a minimum allele frequency of 0.1% and a minimum base quality of 25 Phred-33 scores. The resulting variants were analyzed using RIMA^30^. Variants not overlapping of the cutting window (± 2 base pairs of the cut-site) were excluded. Finally, the editing efficiencies were measured as the percentage of modified reads in mapped reads.

## Supplementary Information

Supplementary Information.

## References

[1] Luyckx VA, Tonelli M, Stanifer JW (2018). The global burden of kidney disease and the sustainable development goals. Bull. World Health Organ..

[2] Levin A (2017). Global kidney health 2017 and beyond: A roadmap for closing gaps in care, research, and policy. Lancet.

[3] Connaughton DM (2015). The Irish Kidney Gene Project-prevalence of family history in patients with kidney disease in Ireland. Nephron.

[4] Vivante A, Hildebrandt F (2016). Exploring the genetic basis of early-onset chronic kidney disease. Nat. Rev. Nephrol..

[5] Webster AC, Nagler EV, Morton RL, Masson P (2017). Chronic kidney disease. Lancet.

[6] O'Seaghdha CM, Fox CS (2011). Genome-wide association studies of chronic kidney disease: What have we learned?. Nat. Rev. Nephrol..

[7] Groopman EE (2019). Diagnostic utility of exome sequencing for kidney disease. N. Engl. J. Med..

[8] Hughes J, Rees S, Kalindjian S, Philpott K (2011). Principles of early drug discovery. Br. J. Pharmacol..

[9] Cook D (2014). Lessons learned from the fate of AstraZeneca's drug pipeline: A five-dimensional framework. Nat. Rev. Drug Discov..

[10] Lancaster MA, Knoblich JA (2014). Organogenesis in a dish: Modeling development and disease using organoid technologies. Science.

[11] Clevers H (2016). Modeling development and disease with organoids. Cell.

[12] Rossi G, Manfrin A, Lutolf MP (2018). Progress and potential in organoid research. Nat. Rev. Genet..

[13] Nishinakamura R (2019). Human kidney organoids: progress and remaining challenges. Nat. Rev. Nephrol..

[14] Kumar, S. V. et al. Kidney micro-organoids in suspension culture as a scalable source of human pluripotent stem cell-derived kidney cells. *Development (Cambridge, England)* **146** (2019). 10.1242/dev.172361 PMC6432662 30846463

[15] Borestrom C (2018). A CRISP(e)R view on kidney organoids allows generation of an induced pluripotent stem cell-derived kidney model for drug discovery. Kidney Int..

[16] Takasato M (2015). Kidney organoids from human iPS cells contain multiple lineages and model human nephrogenesis. Nature.

[17] Morizane R (2015). Nephron organoids derived from human pluripotent stem cells model kidney development and injury. Nat. Biotechnol..

[18] Wiedenheft B, Sternberg SH, Doudna JA (2012). RNA-guided genetic silencing systems in bacteria and archaea. Nature.

[19] Cong L (2013). Multiplex genome engineering using CRISPR/Cas systems. Science.

[20] Mali P (2013). RNA-guided human genome engineering via Cas9. Science.

[21] Lundin A (2020). ObLiGaRe doxycycline Inducible (ODIn) Cas9 system driving pre-clinical drug discovery, from design to cancer treatment. Nat. Commun..

[22] Maresca M, Lin VG, Guo N, Yang Y (2013). Obligate ligation-gated recombination (ObLiGaRe): Custom-designed nuclease-mediated targeted integration through nonhomologous end joining. Genome Res..

[23] Freedman BS (2015). Modelling kidney disease with CRISPR-mutant kidney organoids derived from human pluripotent epiblast spheroids. Nat. Commun..

[24] Cruz NM (2017). Organoid cystogenesis reveals a critical role of microenvironment in human polycystic kidney disease. Nat. Mater..

[25] Geisinger JM, Turan S, Hernandez S, Spector LP, Calos MP (2016). In vivo blunt-end cloning through CRISPR/Cas9-facilitated non-homologous end-joining. Nucleic Acids Res..

[26] Tsai SQ (2015). GUIDE-seq enables genome-wide profiling of off-target cleavage by CRISPR-Cas nucleases. Nat. Biotechnol..

[27] Piontek K, Menezes LF, Garcia-Gonzalez MA, Huso DL, Germino GG (2007). A critical developmental switch defines the kinetics of kidney cyst formation after loss of Pkd1. Nat. Med..

[28] Takasato M (2014). Directing human embryonic stem cell differentiation towards a renal lineage generates a self-organizing kidney. Nat. Cell Biol..

[29] Ye J (2012). Primer-BLAST: A tool to design target-specific primers for polymerase chain reaction. BMC Bioinform..

[30] Taheri-Ghahfarokhi A (2018). Decoding non-random mutational signatures at Cas9 targeted sites. Nucleic Acids Res..

[31] Magoc T, Salzberg SL (2011). FLASH: fast length adjustment of short reads to improve genome assemblies. Bioinformatics.

[32] Li H, Durbin R (2009). Fast and accurate short read alignment with Burrows–Wheeler transform. Bioinformatics.

