# Supplementary material for: Rapid target validation in a Cas9-inducible hiPSC derived kidney model
Source: Sci Rep. 2021 Aug 16;11:16532. doi: 10.1038/s41598-021-95986-5 (PMC8368200; doi:10.1038/s41598-021-95986-5)
Supplement: Supplementary file 1 — Supplementary Information. [file 41598_2021_95986_MOESM1_ESM.docx]

**SUPPLEMENTARY INFO**

**Rapid target validation in a Cas9-inducible hiPSC derived kidney model**


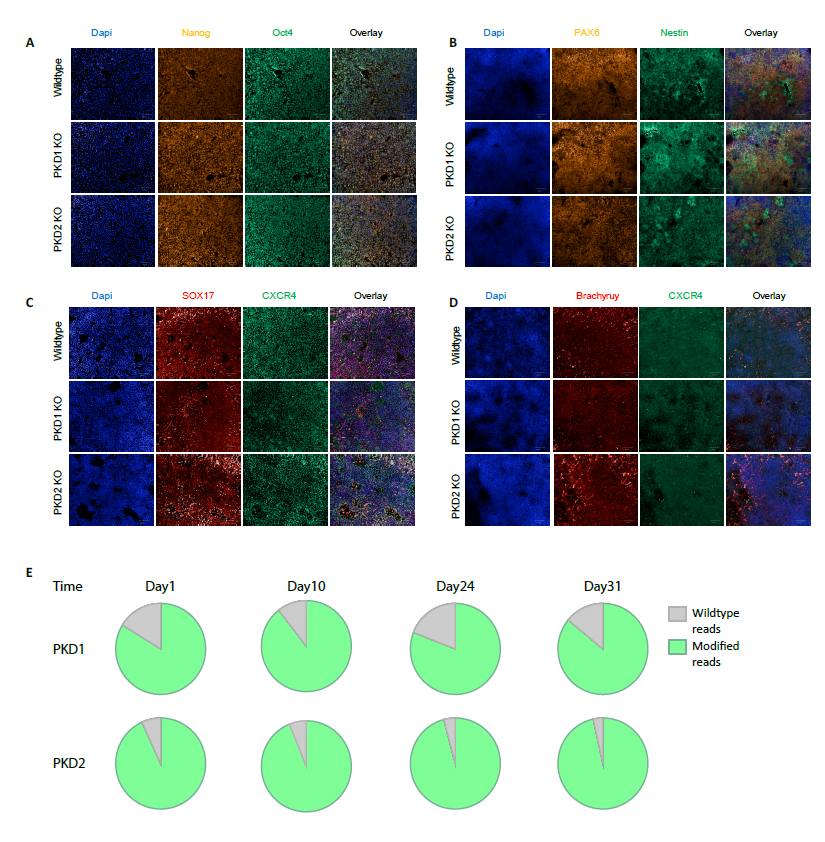


**Supplementary Figure 1.** *PKD1* and *PKD2* are dispensable from pluripotency and tri-lineage differentiation of hiPSCs. **a**) Immunostaining for OCT4 and NANOG in wildtype and PKD knockout pools iPSCs. **b** -**d**) Tri-lineage differentiation of PKD knockout pools. **e**) PKD knockout cells were maintained over the differentiation course.

**Supplementary Table 1.** List of primers used in this study.

| **Primer name** | **Sequence (5´-3´)** | **Gene** | **Used in** |
| --- | --- | --- | --- |
| Yas124 | TCGTCGGCAGCGTCAGATGTGTATAAGAGACAGTGCCTCTCACAGGTCTGTCT |  |  |
| Yas125 | GTCTCGTGGGCTCGGAGATGTGTATAAGAGACAGGGCCTGTAGCCTACCCCT |  |  |
| Yas126 | TCGTCGGCAGCGTCAGATGTGTATAAGAGACAGGATGCAGCGCATCCGGCAG |  |  |
| Yas127 | GTCTCGTGGGCTCGGAGATGTGTATAAGAGACAGTGGTAGCCCCCAAGCCCC |  |  |
| Yas128 | TCGTCGGCAGCGTCAGATGTGTATAAGAGACAGTCTCTGATAGGGCTACCATAAAGC |  |  |
| Yas129 | GTCTCGTGGGCTCGGAGATGTGTATAAGAGACAGTGAGCCCTTGGGCTAGAAAT |  |  |
| Yas185 | TCGTCGGCAGCGTCAGATGTGTATAAGAGACAGATCTCCGGCTCCCAATGAGG |  |  |
| Yas186 | GTCTCGTGGGCTCGGAGATGTGTATAAGAGACAGCGGACTTTCTCATGGACCCT |  |  |
| Yas187 | TCGTCGGCAGCGTCAGATGTGTATAAGAGACAGTTCTCCGCTGCTGTTTCCAC |  |  |
| Yas188 | GTCTCGTGGGCTCGGAGATGTGTATAAGAGACAGGTCGAAGGCTTTCCCGATCA |  |  |
| Yas189 | TCGTCGGCAGCGTCAGATGTGTATAAGAGACAGCAGAGGGTTTGCTAGGTACTCA |  |  |
| Yas190 | GTCTCGTGGGCTCGGAGATGTGTATAAGAGACAGGAAGGAAAGAAACGCGGTGT |  |  |
| Yas191 | TCGTCGGCAGCGTCAGATGTGTATAAGAGACAGTCTCCTTTCCCAGTTGACCG |  |  |
| Yas192 | GTCTCGTGGGCTCGGAGATGTGTATAAGAGACAGACGCTAAATAACTCTGGCCTCC |  |  |
| Yas193 | TCGTCGGCAGCGTCAGATGTGTATAAGAGACAGACCCCACAATCCCCGTCTTC |  |  |
| Yas194 | GTCTCGTGGGCTCGGAGATGTGTATAAGAGACAGTCAGGAACTTTCAGGGGCG |  |  |
| Yas195 | TCGTCGGCAGCGTCAGATGTGTATAAGAGACAGGACTAGGCCGCGATCAGTT |  |  |
| Yas196 | GTCTCGTGGGCTCGGAGATGTGTATAAGAGACAGAGCTTCAATGCGACACAACG |  |  |
| Yas197 | TCGTCGGCAGCGTCAGATGTGTATAAGAGACAGCGGGTTTTCTTCCGGTTGAG |  |  |
| Yas198 | GTCTCGTGGGCTCGGAGATGTGTATAAGAGACAGGCGATAGTGTCCTCGGAACC |  |  |
| Yas199 | TCGTCGGCAGCGTCAGATGTGTATAAGAGACAGAAACAGTTTCGTCCCGAGGC |  |  |
| Yas200 | GTCTCGTGGGCTCGGAGATGTGTATAAGAGACAGGTGGGGGTTCCTTATGACCA |  |  |
| Yas201 | TCGTCGGCAGCGTCAGATGTGTATAAGAGACAGTGGCACAACTTGCTGTGGAG |  |  |
| Yas202 | GTCTCGTGGGCTCGGAGATGTGTATAAGAGACAGGGGTATGGTGACTACGGAGC |  |  |
| Yas203 | TCGTCGGCAGCGTCAGATGTGTATAAGAGACAGCTCCGCATACTTCTTGCCCT |  |  |
| Yas204 | GTCTCGTGGGCTCGGAGATGTGTATAAGAGACAGCTCTTGGCAGCCGCTGAATG |  |  |
| Yas205 | TCGTCGGCAGCGTCAGATGTGTATAAGAGACAGCCAGAAGGGCCTTGAACACT |  |  |
| Yas206 | GTCTCGTGGGCTCGGAGATGTGTATAAGAGACAGTGGGCATTACCTAACGGTGTC |  |  |
| Yas207 | TCGTCGGCAGCGTCAGATGTGTATAAGAGACAGAGTGACCCACAAGAACCAAGG |  |  |
| Yas208 | GTCTCGTGGGCTCGGAGATGTGTATAAGAGACAGCTCAAAGCTGTGTTGGGCCT |  |  |
| Yas209 | TCGTCGGCAGCGTCAGATGTGTATAAGAGACAGACACCATGCAAAAGGCTCTC |  |  |
| Yas210 | GTCTCGTGGGCTCGGAGATGTGTATAAGAGACAGTCAGCAAGTTGACTGTTGACTC |  |  |
| Yas211 | TCGTCGGCAGCGTCAGATGTGTATAAGAGACAGGTCCTCAATGCCACGGAACA |  |  |
| Yas212 | GTCTCGTGGGCTCGGAGATGTGTATAAGAGACAGGACTATGAAGTTGGGCAGCG |  |  |
| Yas213 | TCGTCGGCAGCGTCAGATGTGTATAAGAGACAGGAGCCTTGGAAACGTGGAGG |  |  |
| Yas214 | GTCTCGTGGGCTCGGAGATGTGTATAAGAGACAGTCCACACCGTCTTACCGAGT |  |  |
| Yas215 | TCGTCGGCAGCGTCAGATGTGTATAAGAGACAGAGATCTGATCAGGGCTGGAAC |  |  |
| Yas216 | GTCTCGTGGGCTCGGAGATGTGTATAAGAGACAGGCTGGGTGTCAAGTTCATGTG |  |  |
| Yas217 | TCGTCGGCAGCGTCAGATGTGTATAAGAGACAGGAGATAATGGGTGTGGATCCGT |  |  |
| Yas218 | GTCTCGTGGGCTCGGAGATGTGTATAAGAGACAGATACATTCCCAGGTGCCATGT |  |  |
| Yas219 | TCGTCGGCAGCGTCAGATGTGTATAAGAGACAGGTCCAACCCATGCTGGGTACT |  |  |
| Yas220 | GTCTCGTGGGCTCGGAGATGTGTATAAGAGACAGTTTATTCGTGCAACCCCTCCT |  |  |
| Yas221 | TCGTCGGCAGCGTCAGATGTGTATAAGAGACAGCTTGCTCCCCAAAACGGTTC |  |  |
| Yas222 | GTCTCGTGGGCTCGGAGATGTGTATAAGAGACAGAGGAAGCTGCAGTGGTGATG |  |  |
| Yas223 | TCGTCGGCAGCGTCAGATGTGTATAAGAGACAGGCAAGGTTTGGTGCAGAGTC |  |  |
| Yas224 | GTCTCGTGGGCTCGGAGATGTGTATAAGAGACAGTGACTTTTATGAGAGTTGGTCCTCA |  |  |
| Yas225 | TCGTCGGCAGCGTCAGATGTGTATAAGAGACAGCCCACCCCCATTCAATTGCTTA |  |  |
| Yas226 | GTCTCGTGGGCTCGGAGATGTGTATAAGAGACAGTGGGAAGTAGCAGGGTAGTTT |  |  |
| Yas227 | TCGTCGGCAGCGTCAGATGTGTATAAGAGACAGGTCATCTCTGTTACCTGGGCA |  |  |
| Yas228 | GTCTCGTGGGCTCGGAGATGTGTATAAGAGACAGGGAAGCCCTGAAGAAGGTTG |  |  |
| Yas229 | TCGTCGGCAGCGTCAGATGTGTATAAGAGACAGCCTAATGCCACCAGAGCCTA |  |  |
| Yas230 | GTCTCGTGGGCTCGGAGATGTGTATAAGAGACAGGCCCTTCGGGGAATAATGCT |  |  |
| Yas231 | TCGTCGGCAGCGTCAGATGTGTATAAGAGACAGCACTGTCAGCGACCATAAACT |  |  |
| Yas232 | GTCTCGTGGGCTCGGAGATGTGTATAAGAGACAGCCTGGGAAGTAGCAGGGTAG |  |  |
| Yas233 | TCGTCGGCAGCGTCAGATGTGTATAAGAGACAGAGGCCCTGTTTGTAAGAGTCC |  |  |
| Yas234 | GTCTCGTGGGCTCGGAGATGTGTATAAGAGACAGACCTGGCTCCAGTTTACACC |  |  |
| Yas235 | TCGTCGGCAGCGTCAGATGTGTATAAGAGACAGAAAGCCCAGTTGGGTTCCAT |  |  |
| Yas236 | GTCTCGTGGGCTCGGAGATGTGTATAAGAGACAGACTTCCAAGCCGTATGCTGA |  |  |
| Yas237 | TCGTCGGCAGCGTCAGATGTGTATAAGAGACAGTCTTCCTTGCTACCGCCATC |  |  |
| Yas238 | GTCTCGTGGGCTCGGAGATGTGTATAAGAGACAGGTATGAAGGGCGTACTACCCA |  |  |
| Yas239 | TCGTCGGCAGCGTCAGATGTGTATAAGAGACAGAGAAGTGTCATGAAAATGTGACTGG |  |  |
| Yas240 | GTCTCGTGGGCTCGGAGATGTGTATAAGAGACAGTCAGGTCAAGAAAAATTTGGGGAA |  |  |
| Yas241 | TCGTCGGCAGCGTCAGATGTGTATAAGAGACAGATTTCCCCTTCCCGGATCTGA |  |  |
| Yas242 | GTCTCGTGGGCTCGGAGATGTGTATAAGAGACAGTTCAGCTGATGCCGGTAGAA |  |  |
| Yas243 | TCGTCGGCAGCGTCAGATGTGTATAAGAGACAGTCACACACAAAGAGGGCAGT |  |  |
| Yas244 | GTCTCGTGGGCTCGGAGATGTGTATAAGAGACAGTAGGACTGAGGGTTAGCGGT |  |  |
| Yas245 | TCGTCGGCAGCGTCAGATGTGTATAAGAGACAGGATTTGGTGGACAGCTCAGG |  |  |
| Yas246 | GTCTCGTGGGCTCGGAGATGTGTATAAGAGACAGGGAATGCTGTCCTTTGTGCC |  |  |
| Yas247 | TCGTCGGCAGCGTCAGATGTGTATAAGAGACAGTGCTGGAGAGCCAATGATGG |  |  |
| Yas248 | GTCTCGTGGGCTCGGAGATGTGTATAAGAGACAGTGGTGGGTACCACGGATACA |  |  |
| Yas249 | TCGTCGGCAGCGTCAGATGTGTATAAGAGACAGCTGGAAAGGCACTCAATGGCAA |  |  |
| Yas250 | GTCTCGTGGGCTCGGAGATGTGTATAAGAGACAGTGCACATTTCAGCTGTGAGCTA |  |  |
| Yas251 | TCGTCGGCAGCGTCAGATGTGTATAAGAGACAGTCATGTCTACAGCCCTACTGT |  |  |
| Yas252 | GTCTCGTGGGCTCGGAGATGTGTATAAGAGACAGAAGCATGTGGAACTAGCAAGC |  |  |
| Yas253 | TCGTCGGCAGCGTCAGATGTGTATAAGAGACAGACATTTGAATGCAATTATGGAGGCT |  |  |
| Yas254 | GTCTCGTGGGCTCGGAGATGTGTATAAGAGACAGGCATCTTTGTAGGACACAGGC |  |  |
| Yas255 | TCGTCGGCAGCGTCAGATGTGTATAAGAGACAGAAGGGCTTGTCACAGTCCAG |  |  |
| Yas256 | GTCTCGTGGGCTCGGAGATGTGTATAAGAGACAGTCAGAACTGGAGATGAGCAATGA |  |  |

Note: the NGS linker sequences are highlighted in yellow and green for forward and reverse primers, respectively.
